# Recycling of diaper wastes for a triboelectric nanogenerator-based weather station

**DOI:** 10.1016/j.isci.2024.110627

**Published:** 2024-07-31

**Authors:** Sayyid Abdul Basith, Ananthakumar Ramadoss, Gaurav Khandelwal, George Jacob, Arunkumar Chandrasekhar

**Affiliations:** 1 Nanosensors and Nanoenergy Lab, Biomedical Instrumentation Lab, Department of Sensor and Biomedical Technology, School of Electronics Engineering, Vellore Institute of Technology, Vellore, Tamil Nadu 632014, India; 2 Advanced Research School for Technology & Product Simulation (ARSTPS), School for Advanced Research in Petrochemicals (SARP), Central Institute of Petrochemicals Engineering & Technology (CIPET), T.V.K. Industrial Estate, Guindy, Chennai 600032, India; 3 James Watt School of Engineering, University of Glasgow, Glasgow G12 8QQ, Scotland, UK; 4 Centre for Nanotechnology Research, Vellore Institute of Technology, Vellore, Tamil Nadu 632014, India

**Keywords:** Engineering, materials science, electronic materials

## Abstract

Escalating concerns over waste management and the need for sustainable energy have prompted innovative solutions at the nexus of resource recycling and self-powered applications. This study presents a novel approach to recycling super-absorbing polymer (SAP) gels from waste diapers and discarded baking sheets to fabricate a diaper waste-based triboelectric nanogenerator (DW-TENG). The DW-TENG, resembling a maraca, demonstrated superior electrical performance with a voltage output of 110 V, a current of 9 μA, and a power of 259.15 μW. It was successfully integrated into a self-powered weather station for real-time monitoring of wind speed, humidity, and temperature. This research underscores the dual benefits of waste management and energy generation, representing a promising step toward a circular and sustainable future.

## Highlights

• Transforms diaper and baking sheet wastes into sustainable energy sources
• Uses triboelectric nanogenerator technology for energy harvesting from waste
• Demonstrates a self-powered weather station using recycled waste materials
• Addresses global waste management challenges, promoting a circular economy

## Graphical abstract

**Figure:**
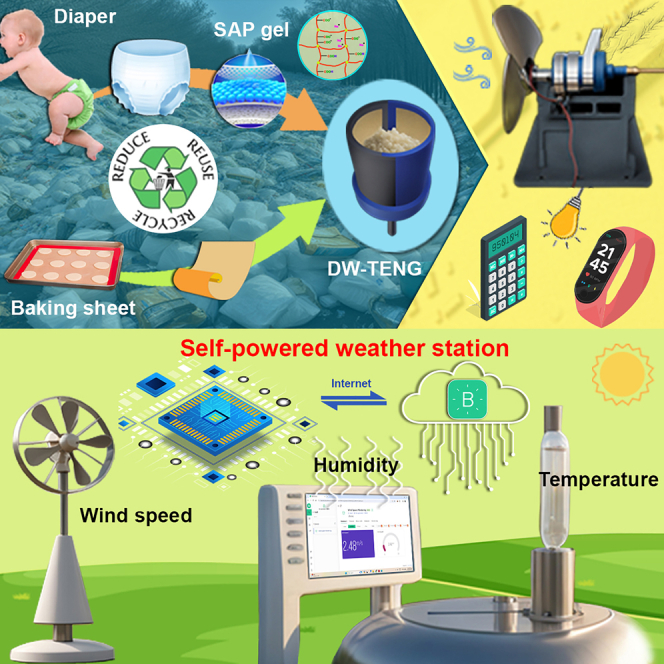

## Introduction

The increasing global concern over waste management and the need for sustainable energy sources have propelled innovative approaches toward resource utilization and energy generation.^1^ One such creative endeavor involves recycling super-absorbing polymer (SAP) gel obtained from discarded diapers and used non-stick baking sheets for dual purposes: waste reduction and renewable energy harvesting. This study presented a novel application of triboelectrification principles, contact electrification,^2^ and electrostatic induction^3^ to convert these wastes into a source of self-generated electricity.^4^^,^^5^ The focus is on fabricating and applying a novel diaper waste-based triboelectric nanogenerator (DW-TENG) designed for anemometer functionality and wind energy harvesting.

Diapers are ubiquitous in modern society, especially in developed and urbanized regions, due to their convenience and high absorbency.^6^^,^^7^ According to the United Nations Environment Programme (UNEP) 2023 report, around 250 million single-use diapers are discarded globally every day. These diapers often end up in landfills or are incinerated, taking up to 500 years to decompose, as estimated by the Environmental Protection Agency (EPA) 2021 report.^8^ The non-biodegradable components, including polypropylene, polyester, stearyl alcohol, thermoplastic polymer, polyacrylic acid, and fragrance, pose significant environmental and health challenges.^9^

**Figure 1:**
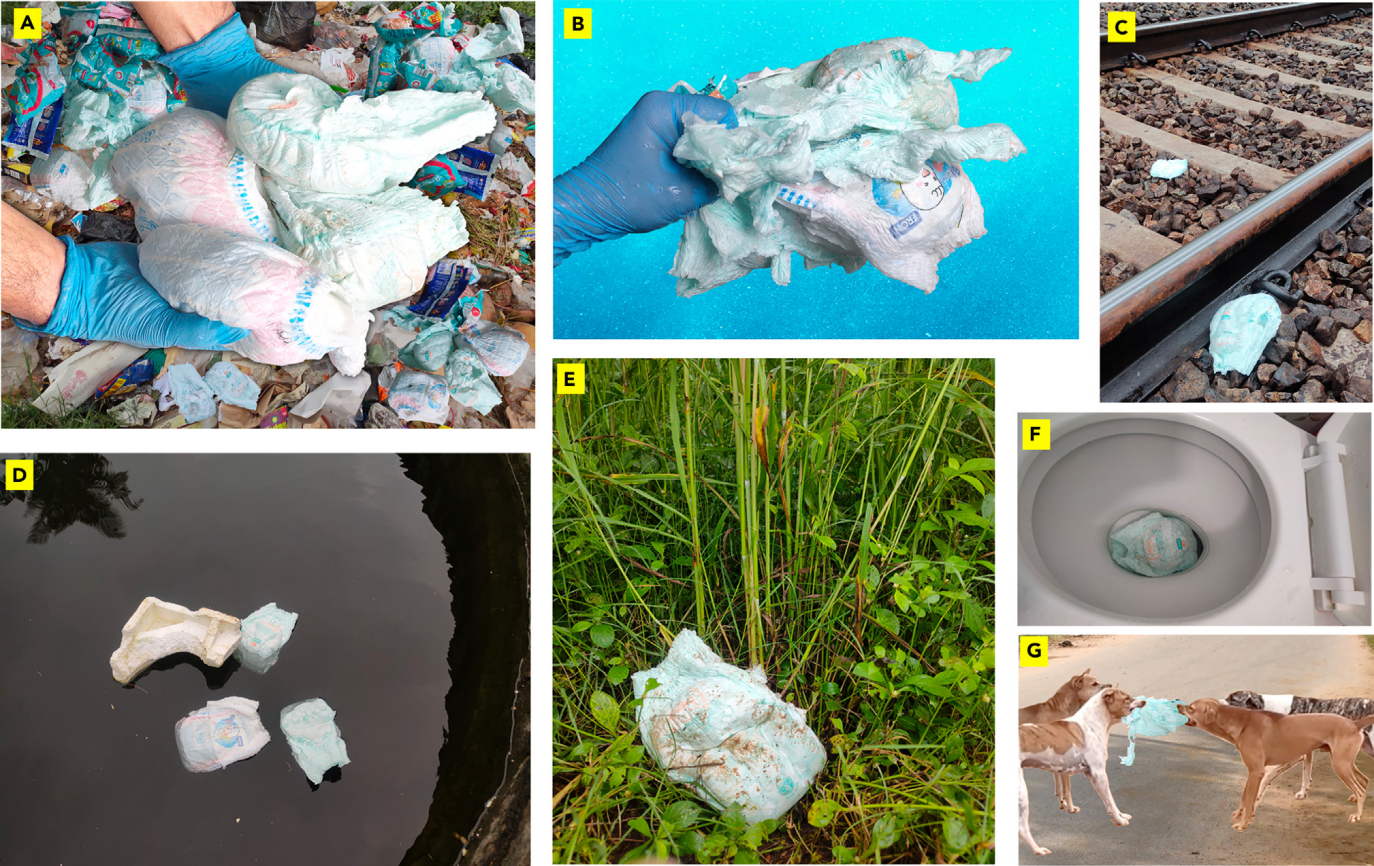
Environmental pollution caused by disposed diapers in various fields (A) Landfills. (B) Ocean. (C) Railway tracks. (D) Public ponds. (E) Agricultural farms. (F) Public toilets. (G) Dogs fighting with diapers.

Disposed diapers can spread infectious diseases and harbor pathogens such as bacteria (e.g., *Escherichia coli* and *Staphylococcus aureus*),^10^^,^^11^ viruses (e.g., norovirus and rotavirus),^12^ and parasites (e.g., *Giardia lamblia* and *Cryptosporidium*).^13^ These pathogens pose health risks, especially in moist environments where fungi like *Candida albicans* can also thrive.^14^ Additionally, the super absorbent quality of diapers can contaminate groundwater.^15^ Rainwater infiltrating landfill waste generates leachate—a liquid mixture of substances from decomposing diapers, including SAP particles.^16^ Without proper management, this leachate may permeate the soil, potentially reaching groundwater and introducing SAPs into the water table.^17^ Some pathetic visuals of the environmental pollution issues caused by the waste diapers to landfills, oceans, public transport, water resources, agricultural farms, and animal lives are illustrated in Figure 1.

Several methods have been proposed for recycling waste diapers to mitigate their environmental impact. Trilokesh et al. developed a way to recycle baby diaper waste into cellulose and nanocellulose using a chlorine-free approach, extracting approximately 25.13% w/w cellulose from used diapers.^18^ Wang et al. described a method for recycling diaper waste into a magnetic catalyst for glycerol carbonate synthesis by calcinating diaper waste above 400°C after impregnating it in nickel nitrate solution.^19^ Budyk et al. removed water from disposable diapers and produced high-calorific hydrochar using hydrothermal carbonization, resulting in better combustion characteristics and higher carbon content than dry diapers.^9^ Itsubo et al. developed a technology for closed-loop recycling of used paper diapers, evaluating the use of recycled pulp and SAP as materials via life cycle assessment (LCA).^20^ Espinosa-Valdemar et al. demonstrated that composting used baby diapers with yard waste is a feasible and affordable valorization option.^21^

In contrast, the proposed DW-TENG introduces a novel and sustainable approach by harnessing the mechanical energy from diaper waste for energy harvesting, offering a dual solution to waste management and energy generation. In this work, for the first time, naturally dried SAP gels from sterilized waste diapers are used to fabricate a triboelectric nanogenerator (TENG). Environmental impacts associated with the diaper waste recycling process are discussed in the supplemental information.

The used baking sheets obtained from landfills serve as the second active layer of the TENG. The non-stick heat-resistant baking sheets are specialized cooking and baking accessories made by coating polytetrafluoroethylene (PTFE) onto a fabric substrate.^22^ These sheets feature a smooth, non-stick surface that prevents food from adhering to them during cooking or baking. PTFE coating is renowned for its exceptional non-stick properties, high resistance to heat up to 260°C, and chemical inertness, making it a versatile choice in the kitchen. However, PTFE is a synthetic, non-biodegradable fluoropolymer that can generate microplastics if it reaches the environment. The incineration of PTFE or PTFE-coated materials can release toxic fumes and gases, including perfluorinated compounds.^23^ While much effort has not been made to recycle PTFE-coated baking sheets, several approaches, including hydrothermal treatment,^24^ solvent-based methods,^25^ physical separation,^26^ and depolymerization^27^ have been proposed to break down or separate PTFE coating from the fabrics. In this work, these PTFE-coated baking sheets are directly reused for the fabrication of TENG after cleaning and disinfection.

The naturally dried SAP powder and baking sheet were recovered through meticulous sterilization. They are then transformed into usable forms as positive and negative triboelectric materials for the fabrication of maraca-shaped DW-TENG. By exploiting the electrostatic properties of SAP powder and baking sheets and based on triboelectrification between these materials, this work was able to collect 259.15 μW of electric power by simple shaking of DW-TENG and build a self-powered mini weather station. This work aligns with the Sustainable Development Goals (SDGs) 3, 7, and 11. This research embarks on a transformative journey to mitigate the environmental impact of disposable diapers. SDG 3’s goal of good health and well-being is supported through environmentally friendly practices in waste management. SDG 7’s emphasis on affordable and clean energy is met by introducing the DW-TENG as an innovative energy harvester. Furthermore, SDG 11, focusing on sustainable cities and communities, is indirectly addressed by offering a solution for urban waste management.^28^

To provide a comprehensive understanding of the performance, novelty and significance of our DW-TENG, we compared its key metrics with those of recently reported TENGs fabricated from various recycled materials.^29^^,^^30^^,^^31^^,^^32^^,^^33^^,^^34^^,^^35^^,^^36^^,^^37^ In literature, Jiang et al. investigated triboelectric properties of natural polymers such as egg white (EW), chitin derived from crab and shrimp shells, silk fibroin (SF), and rice paper (RP) and developed a biodegradable implantable TENG for *in vivo* cardiovascular and neurological disease therapy.^38^ Alluri et al. utilized aloe vera leaves as a triboelectric positive material to harvest mechanical energy in contact separation mode.^39^ Ma et al. observed that the bladder film of grass carp fish has a triboelectric positive charge affinity due to the presence of collagen fibers on its surface and demonstrated a single-electrode mode TENG for non-contact position monitoring sensor.^40^ Basith et al. recycled surgical three-layer face masks and nitrile hand gloves to fabricate a self-powered touch sensor for IoT applications.^41^ In another work by Basith et al. Polyethylene terephthalate (PET) sheets were utilized as a supporting framework for developing a contact separation TENG and demonstrated its effectiveness in a completely battery-free tally counter application.^42^ Zhang et al. reused nylon cloths as triboelectric positive material and Polyvinyl chloride (PVC) carry covers as negative material for a Morse code generator for emergency conditions.^43^ Rani et al. recycled cigarette filters to fabricate a contact separation TENG for energy harvesting applications.^44^

In our study, we have utilized SAP gels obtained from discarded diapers combined with non-stick baking sheets to fabricate a TENG, DW-TENG. This combination of materials is novel and has not been extensively explored in TENG research, providing a fresh perspective on material selection for triboelectric applications. The DW-TENG achieved a voltage output of 110 V, a current of 9 μA, and a power of 259.15 μW/cm^2^. Compared to the aforementioned previous works, the DW-TENG demonstrates superior electrical performance. The fabricated DW-TENG also demonstrates its capability as a self-powered weather station to monitor real-time environmental conditions such as wind speed, atmospheric temperature, and humidity through the support of IoT. This highlights its practical applications in real-world scenarios beyond mere energy harvesting, showcasing its versatility and utility. A performance comparison of these recently reported TENGs with the proposed DW-TENG is given in Table 1.

**Table 1:** Comparison of recently reported TENGs fabricated from various recycled materials

Type of Waste	Mode of transformation	Functionality in TENG	Mode of operation	Output performances			Application
				V_oc_ (V)	I_sc_ (μA)	P (μW)	
SF, RP, EW, cellulose and chitin^38^	ICP etching and methanol treatment	Positive and negative materials	Contact separation	55	0.6	21.6	*In vivo* disease therapy
Aloe vera leaf^39^	Spin-coating process	Positive material	Contact separation	1.2	0.22	0.42	Energy harvesting
Fish bladder film^40^	Cleaning and drying	Positive material	Single electrode	106	7.3	390	Position monitoring
Surgical face mask and nitrile glove^41^	Disinfection and Sterilization	Positive and negative materials	Contact separation	50.7	4.8	57.5	Touch sensor
PET bottles^42^	Cleaning and sterilization	Supporting framework	Contact separation	90	8.2	200	Battery-free tally counter
PVC carry cover and nylon cloths^43^	Directly used after cleaning and drying	Positive and negative materials	Contact separation	31	3.1	11	Morse code generator
Cigarette filter^44^	Directly used after cleaning and drying	Positive materials	Contact separation	42.8	0.86	63.2	Energy harvesting
Diaper SAP gel and baking sheets (This work)	Sterilization using ethanol, heating, and UV treatment	Positive and negative materials	Freestanding mode	110	9	259.15	Rotational Energy harvester and self-powered weather station

## Results and discussion

### Materials and methods

**Figure 2:**
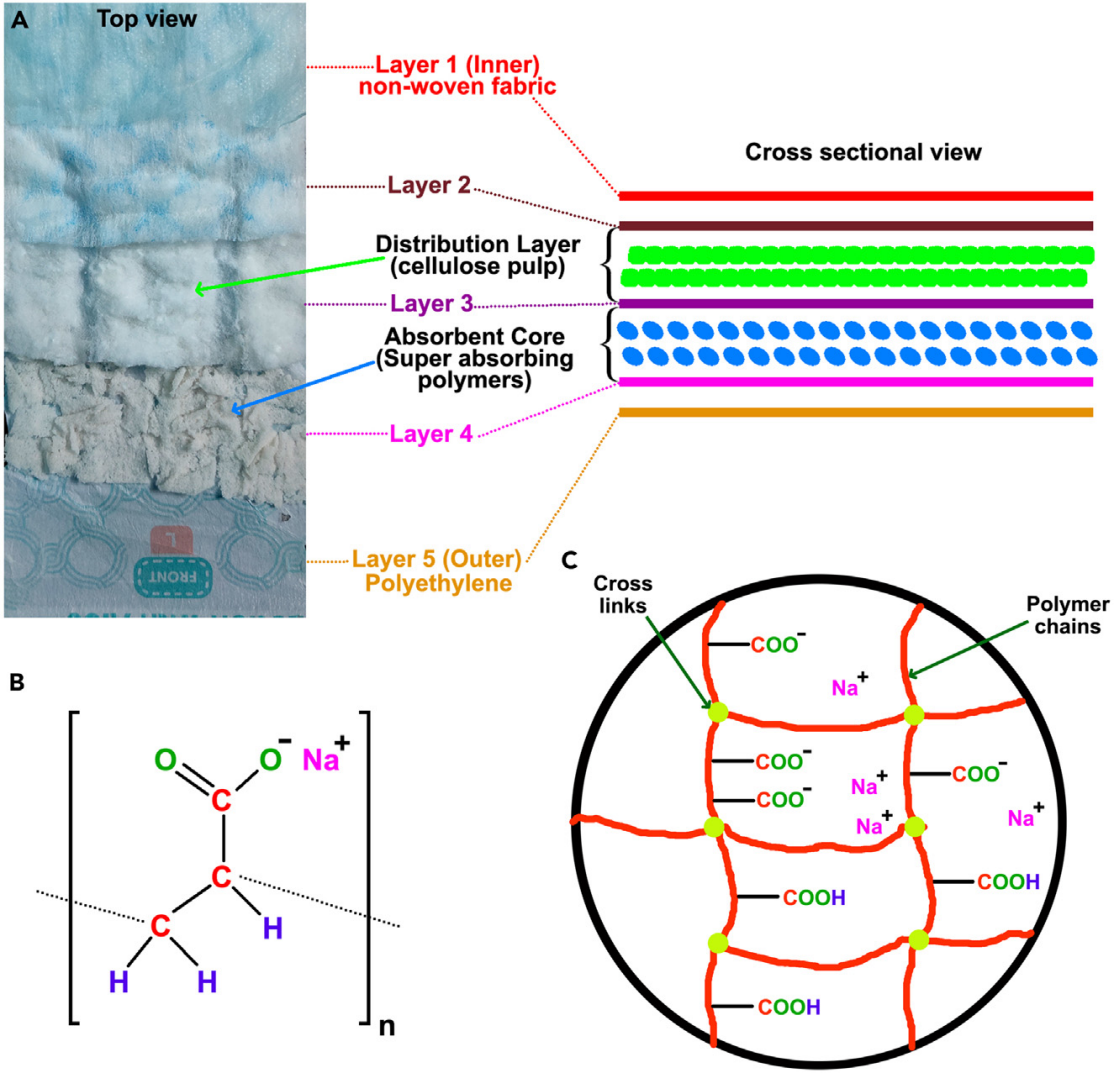
Structure of diaper and SAP (A) Diaper functional layers. (B) Chemical structure of SAP. (C) Cross-linked polymer network of SAP.

The core materials for fabricating DW-TENG are the disposed diaper gels and baking sheets. The primary function of a diaper is to absorb and retain urine. A typical disposable diaper consists of five layers designed to provide absorbency and leak prevention^45^ (Figure 2A). The inner layer (layer 1), which comes into direct contact with the baby’s skin, is made of a non-woven fabric that is designed to keep the skin dry and comfortable. Between layers 2 and 3, the distribution layer is filled with cellulose pulps, which help to distribute the absorbed liquid evenly throughout the core, ensuring efficient absorption. The absorbent core between layers 3 and 4 is the core of the diaper, which is filled with SAP and is responsible for absorbing and retaining urine. Finally, it has an outer shell (layer 5) made of waterproof polyethylene to prevent leaks. It serves as a barrier between the absorbent core and the outside environment.

The SAP in diapers, primarily composed of sodium polyacrylate, is a cross-linked polymer with hydrophilic carboxylate groups (-COO^−^) that enable high water absorption (Figures 2B and 2C).^46^^,^^47^ When in contact with water, the SAP forms a gel, capable of absorbing up to 400 g of water per gram of dry polymer, without dissolving.^48^^,^^49^ The water remains trapped within the polymer structure until external pressure or conditions cause it to be released.^50^^,^^51^

**Figure 3:**
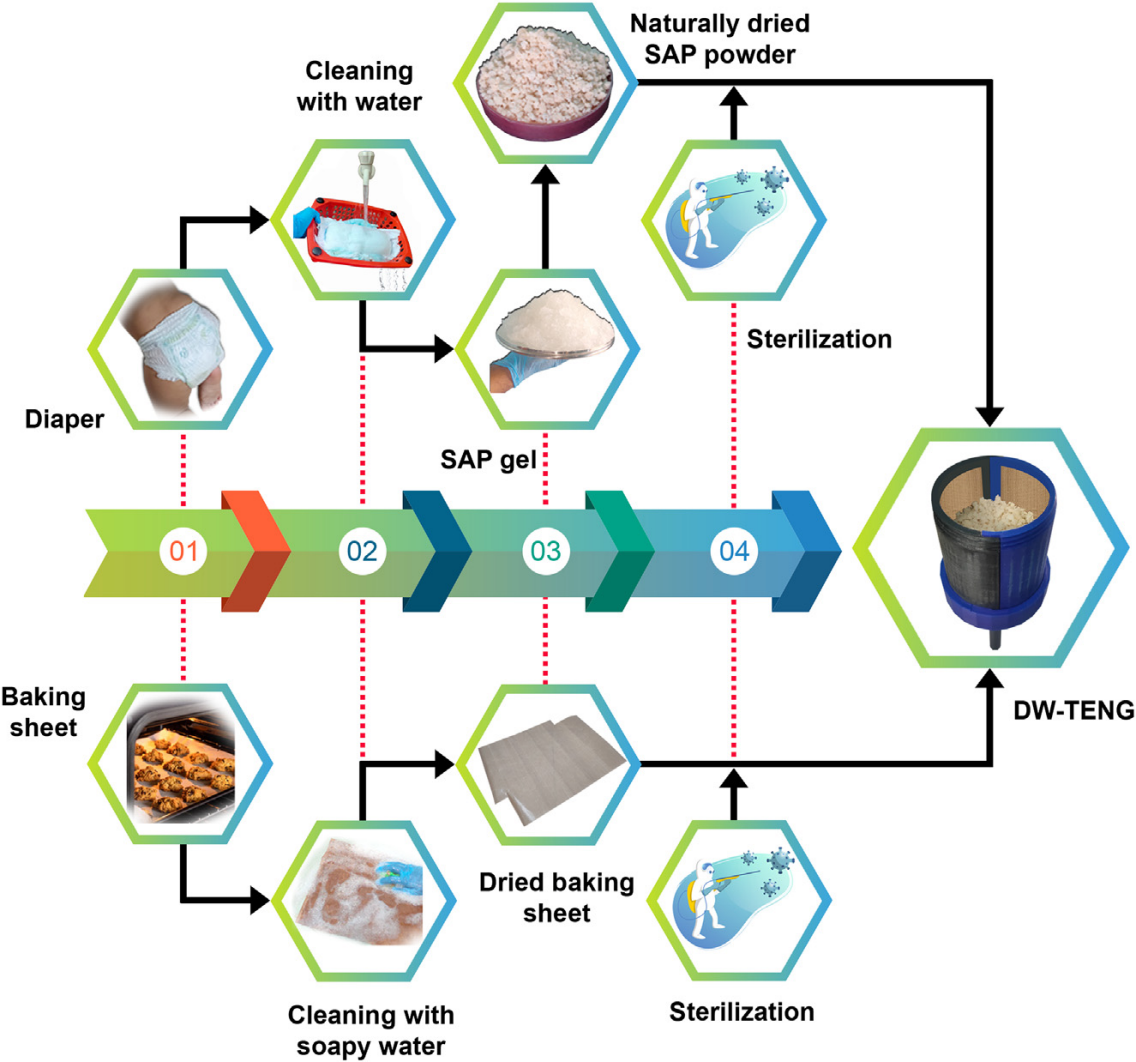
Workflow of DW-TENG fabrication from waste diapers and baking sheets

The absorption of urine by SAP is a reversible process.^52^ This reversibility allows for the controlled release of trapped water when needed. Recovering SAP from urinated diapers undergoes several steps. Initially, the urinated diapers were soaked in clean water and gently squeezed to release as much liquid as possible. Then, the squeezed SAP gels were collected from the diaper and rinsed with clean water to help remove the urine and contaminants. A large volume of water was used to agitate the gel during the rinsing. This step was repeated several times until the water ran clearly. The water from the SAP gel was drained and it was squeezed again to remove excess moisture. It was ensured that the applied pressure was low, as high pressure can damage the gel or the polymer chains. Figure S1 shows the cleaning test conducted on urine-soaked diaper gels in the lab with multiple rinses with clean water. After rinsing and squeezing, SAP gel was laid in a well-ventilated area without exposure to sunlight for one week to evaporate the remaining water. A day-by-day analysis of the natural drying of SAP gels for seven days is shown in Figures S2A–S2G. Finally, the dried SAP gel was gently crushed to break it into smaller particles to get dried SAP in powder form, as shown in Figure S2H. Similarly, baking sheets obtained from landfills were well-cleaned using soapy water and dried in air. Both the materials, SAP powder and baking sheet, underwent sterilization and sterility tests to confirm their reusability (see STAR methods). A complete workflow toward DW-TENG fabrication is graphically illustrated in Figure 3.

### Characterization techniques

**Figure 4:**
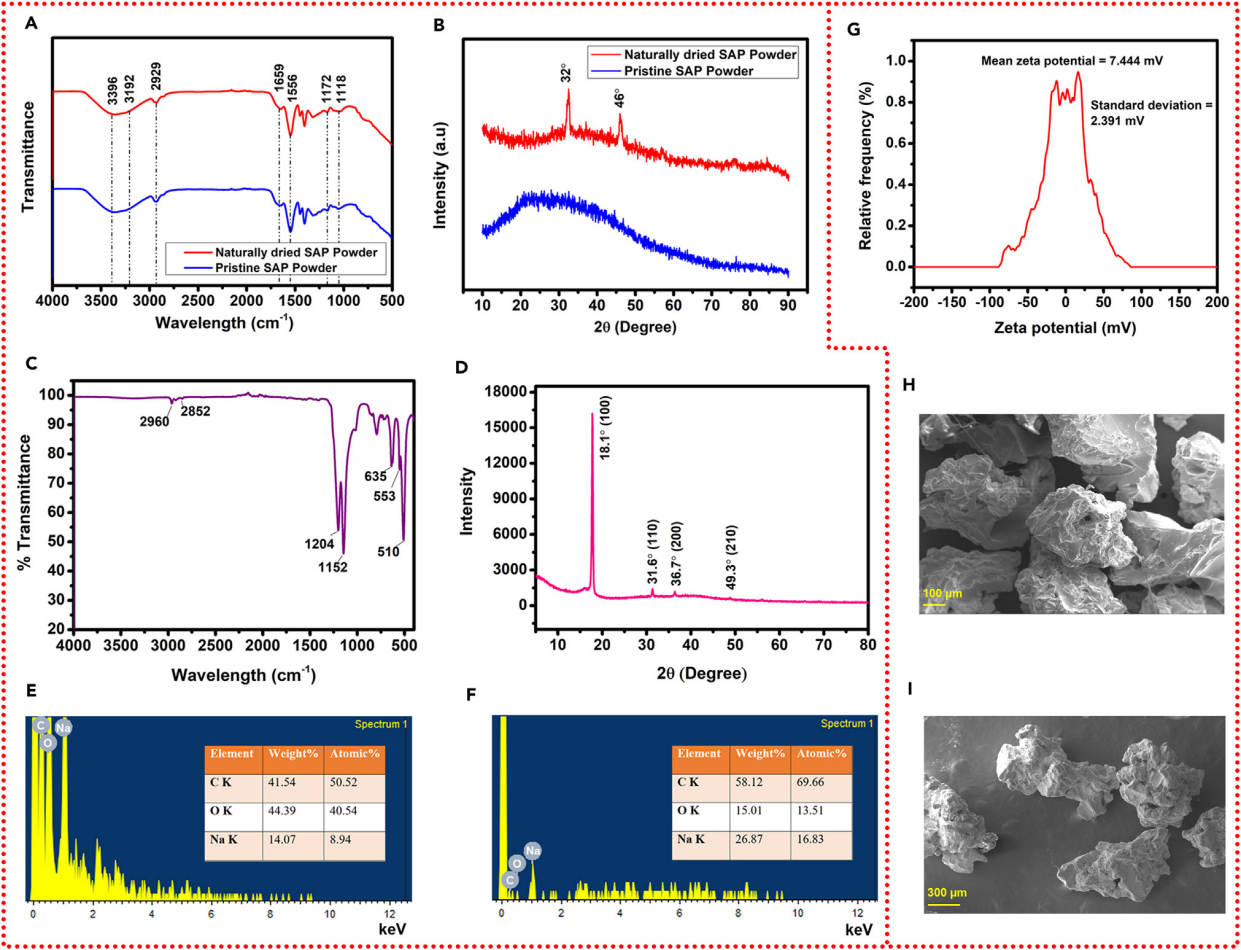
Characterization results (A) FTIR and (B) XRD spectrum comparison between naturally dried SAP powder and pristine SAP. (C) FTIR and (D) XRD spectrum of the baking sheet. (E) EDX spectra of naturally dried SAP. (F) EDX spectra of pristine SAP. (G) Zeta potential plot of naturally dried SAP powder. (H) SEM images of pristine SAP. (I) SEM images of naturally dried SAP.

Various characterization techniques were employed to comprehensively understand the structural and chemical properties of the key components involved in the DW-TENG. The detailed characterization sets the foundation for understanding the materials’ suitability and performance in the DW-TENG. Fourier transform infrared spectroscopy (FTIR) and X-ray diffraction (XRD) analyses were conducted on the finally obtained naturally dried SAP powder to characterize its structural and chemical properties and compared its spectra with the FTIR and XRD of the pristine SAP powder collected from the non-urinated and non-dumped diapers, as shown in Figures 4A and 4B. FTIR spectra analysis of naturally dried SAP powder helps to understand the formation and cross-linking of the sodium polyacrylate SAP. In the spectrum, the peaks observed at 3,396 cm^−1^ and 3,192 cm^−1^ correspond to O–H hydroxyl and N–H amide stretching, respectively. The absorbance at 2,929 cm^−1^ is assigned to the –C–H stretching of the acrylate group. The peak at 1,659 cm^−1^ is assigned to the carbonyl (–C=O) group. The absorbance at 1,556 cm^−1^ is attributed to the sodium acrylate –COONa group, and the peak at 1,172 cm^−1^ corresponds to the carboxylate –COO⁻ group. The spectrum of naturally dried SAP matches the FTIR of the pristine SAP, revealing the existence of carboxylate functional groups after drying the SAP powder, and these functional groups are part of the polymer’s chemical structure.^53^ Drying involves the removal of water from the material, but it does not typically lead to chemical changes in the polymer’s backbone or its functional groups.

The broad scattering pattern without distinct peaks in the pristine SAP powder XRD pattern represents sodium polyacrylate’s amorphous nature. This amorphous nature of sodium polyacrylate contributes to its ability to absorb and retain large amounts of water.^54^ However, the XRD pattern of naturally dried SAP powder shows the presence of distinct peaks at 32º and 46º, which reveals the formation of some level of crystallinity in the naturally dried SAP. The formation of this crystallinity indicates that the drying process led to the arrangement of more ordered polymer chains, resulting in a partially crystalline structure. The appearance of crystallinity in dried SAP has affected its reabsorption properties.

FTIR of the baking sheet, as plotted in Figure 4C, shows the stretches at ∼2,960 cm^−1^ and ∼2,852 cm^−1^ and the characteristic absorption peaks at wavelengths 1,204 cm^−1^, 1,152 cm^−1^, 635 cm^−1^, 553 cm^−1^, and 510 cm^−1^. The FTIR spectra of the baking sheet match well with the FTIR spectra of PTFE reported in literature.^55^ Similarly, the XRD pattern of the baking sheet is shown in Figure 4D, which shows peaks at 18.1°, 31.6°, 36.7°, and 49.3°. These peaks are attributed to the (100), (110), (200), and (210) peaks and match with the XRD spectra of PTFE.^56^ Hence, the FTIR and XRD spectra analysis confirmed the presence of PTFE coating over the baking sheet. Energy-dispersive X-ray spectroscopy (EDS) analysis of both the pristine SAP powder and naturally dried SAP powder are shown in Figures 4E and 4F, which provides information about their morphology and material elemental composition. EDS confirmed the presence of carbon (C), oxygen (O), and sodium (Na) in both the SAPs. The relative concentrations of material elements in the dried state were 58.12 weight% of C (69.66 atomic%), 15.01 weight% of O (13.51 atomic%), and 26.87 weight% of Na (16.83 atomic%).

Zeta potential analysis of the naturally dried SAP powder was carried out to understand its surface charge characteristics by creating a stable suspension of the naturally dried SAP powder dispersed in ethanol. A mean zeta potential value of 7.444 mV with a standard deviation of 2.391 mV was observed using a Litesizer 500 zeta potential analyzer instrument, as shown in Figure 4G. The positive zeta potential indicates a net positive static charge on the surface of the dried SAP powder.^57^^,^^58^ Hence, dried SAP powder was selected as the triboelectric positive material for DW-TENG fabrication. This observation is supported by the use of polyacrylate balls as triboelectric positive material while fabricating a multi-directional water wave and vibration energy harvester.^59^ The baking sheet is coated with PTFE, which is a well-reported negative triboelectric material.^60^^,^^61^ Scanning electron microscopy (SEM) images of both the naturally dried SAP powder and pristine SAP powder are shown in Figures 4H and 4I, and High-resolution optical microscopic visual of the baking sheet and its surface morphology analysis using SEM are given in Figures S3A and S3B. The optical microscopic visual of the baking sheet shows the woven structure of the fabric used to make it, and the plain surface morphology by SEM reveals the presence of PTFE coating over the fabric material.

### Fabrication of DW-TENG

**Figure 5:**
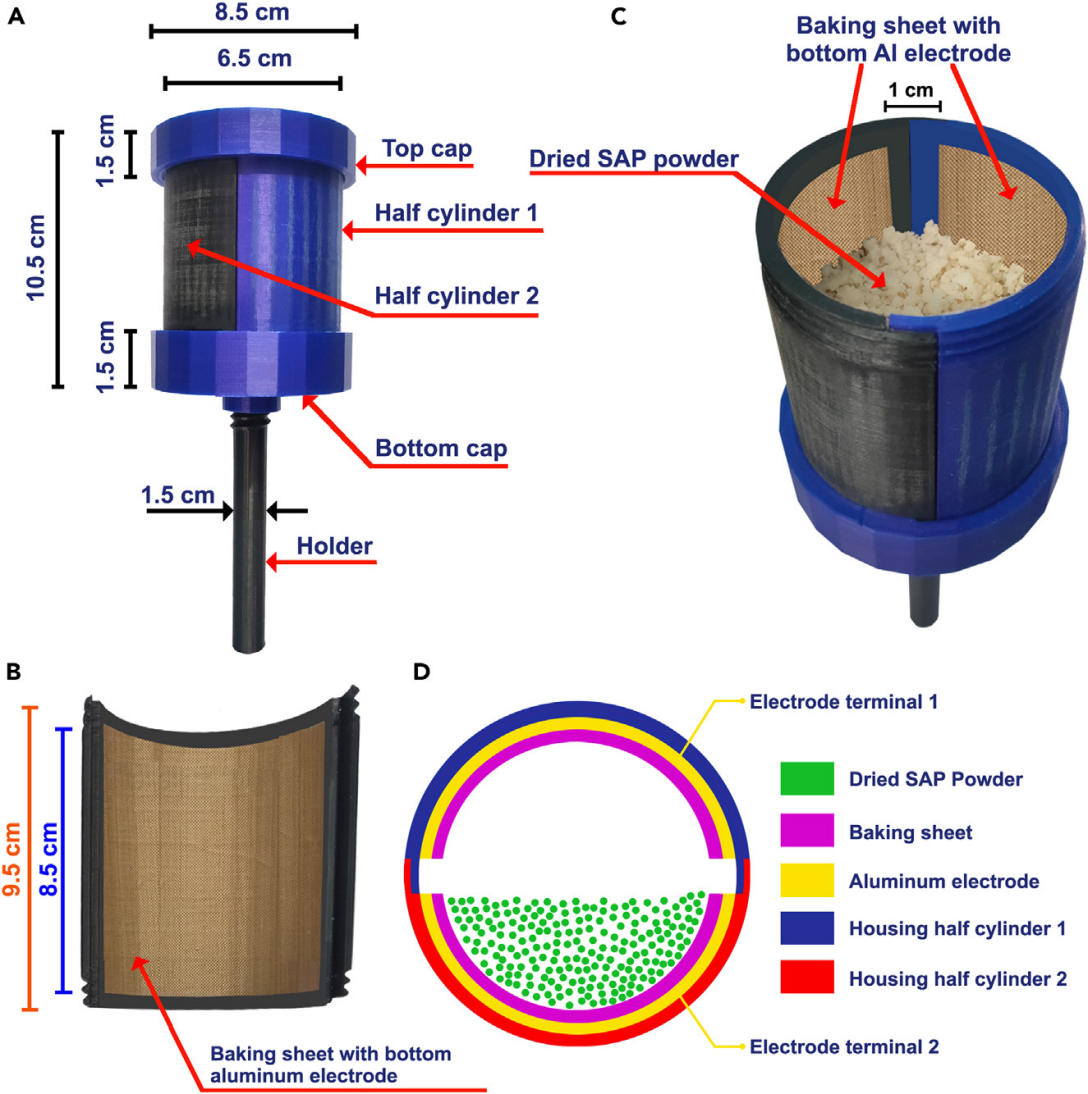
Structure of DW-TENG (A) Outside view with dimensions. (B) Aluminum-coated baking sheet fixed inside of the vertical half-cylinder. (C) DW-TENG filled with dried SAP powder and side walls with aluminum coated baking sheet. (D) Cross-sectional view of DW-TENG.

The DW-TENG was fabricated based on the triboelectrification between the naturally dried SAP powder and the baking sheet. A cylindrical-shaped housing unit for the DW-TENG, reminiscent of a maraca, was fabricated using polylactic acid (PLA) material using 3D-printing technology. The housing unit comprises five integral components: two vertical half-cylinders, one top cap, one bottom cap, and one holder, as shown in Figure 5A.

The core components in DW-TENG are the dried SAP powder and baking sheet. A pair of disinfected rectangular-shaped baking sheets with dimensions 8 cm × 8.5 cm were taken, and the back side of each was coated with aluminum. The aluminum-coated baking sheets were fixed within each of the vertical half-cylinders of the 3D-printed container (Figure 5B). Here, these pairs of aluminum act as two electrodes for DW-TENG. After fixing these electrode-coated baking sheets, the vertical half-cylinders were joined together, and the lower end was sealed using the bottom cap. The dried and sterilized SAP powder was then filled into the 3D-printed cylindrical container up to approximately 75% of the container volume (Figure 5C). The assembly was completed by attaching the upper cap to the open end of the cylindrical container, effectively enclosing the dried SAP powder within. A cross-sectional view of DW-TENG is shown in Figure 5D. 0.1 mm low gauge wires were used to pull out electrode terminals from each aluminum electrode by inserting a small hole in each half cylinder. These electrode terminals assisted the device in making connections with external circuits and devices. The threaded upper and lower caps seal the container, ensuring a secure and tight fit. Finally, the holder is affixed to the bottom cap, allowing to shake the device manually. When joined, the vertical half-cylinders form a cylindrical container with dimensions of 6.5 cm in diameter and 10.5 cm in height.

As the device was shaken, the SAP powder came into intermittent contact with the baking sheets, initiating charge separation and enabling a continuous flow of electric current through the circuit between the aluminum electrodes. This meticulously designed and fabricated device represents a novel approach to harnessing energy from recycled materials, providing a sustainable and eco-friendly avenue for self-powered applications.

### Working mechanism

The heart of this innovative energy-harvesting device lies in the synergistic interaction between dried SAP powder and baking sheets. This interaction leverages the principles of triboelectrification and electrostatic induction, facilitating the conversion of mechanical energy into electrical energy. As the device is manually shaken or agitated, the SAP powders come into intermittent contact with the surface of baking sheets within the 3D-printed cylindrical container. This contact initiates a process known as contact electrification. Due to the inherent electrostatic properties of these materials, surface charge transfer occurs between them at the molecular level. The SAP powders, characterized by a positive affinity for electrons, transfer electrons to the surface of baking sheets, which exhibit a negative charge affinity due to the presence of PTFE over the surface. This transfer of electrons results in the accumulation of a positive charge on the SAP powder particles and a negative charge on the baking sheets.

**Figure 6:**
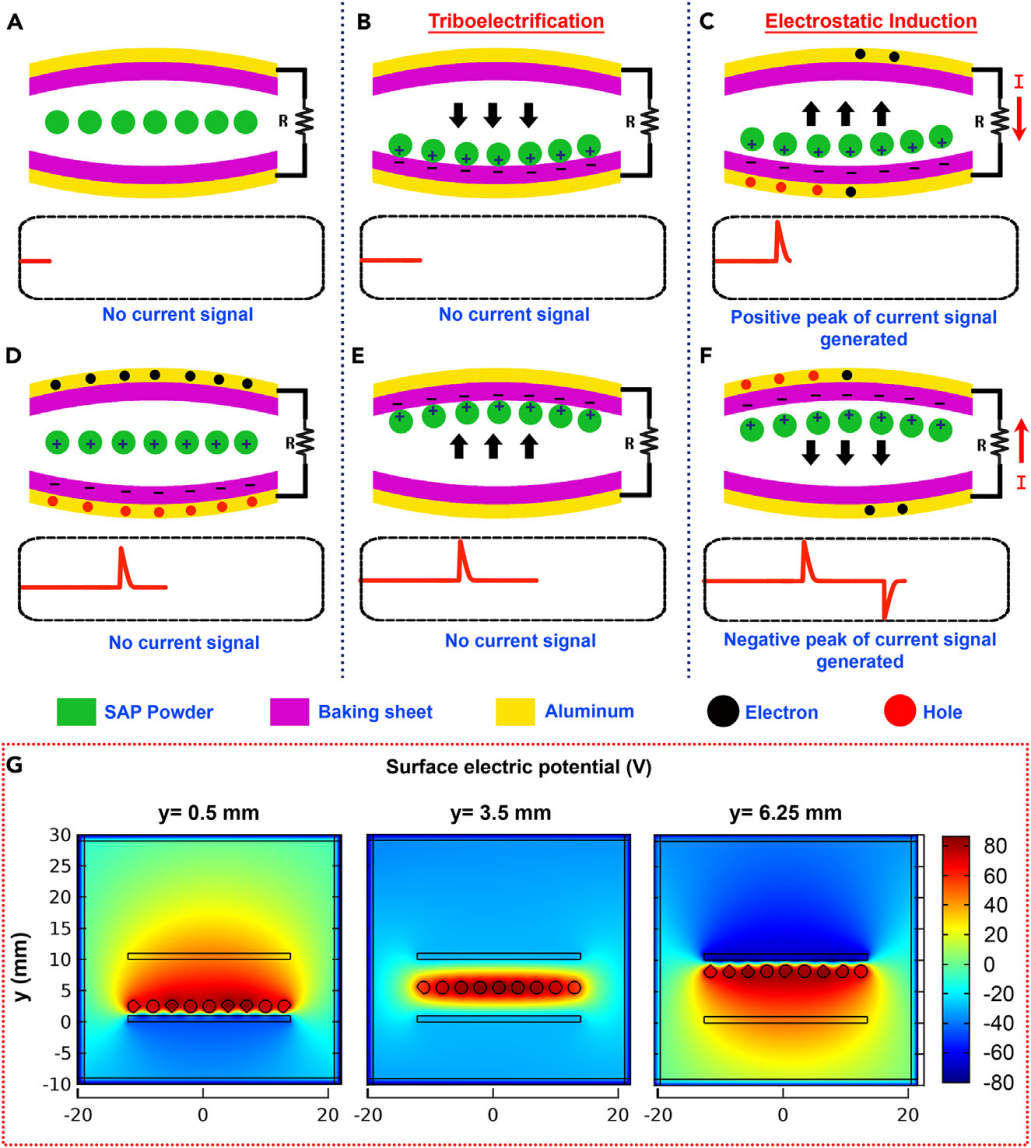
Working mechanism (A–F) Electricity generation in DW-TENG. (G) Surface electric potential distributions obtained by COMSOL simulation at different y positions.

While separating SAP from the baking sheet surface, the aluminum attached to the baking sheet becomes induced with positively charged holes due to their contact with the negatively charged baking sheet. At the same time, the counter aluminum electrode becomes negatively charged with electrons. Similarly, the polarity reversal on aluminum electrodes happens when the same SAP powders come into contact with the baking sheet on the other side of the cylinder. In such a way, the cyclic and repeated shaking of the device ensures the alternate polarity changes in the aluminum electrodes, causing a continuous flow of sinusoidal AC electric current through the external circuit established between the aluminum electrodes (Figures 6A–6F).

The strong electronegativity of fluorine atoms in PTFE causes a significant attraction and retention of electrons for an extended period on its surface.^62^^,^^63^^,^^64^ This results in a higher accumulation of negative charges on the PTFE surface through contact electrification. Consequently, this increased charge density on the PTFE enhances the induction of opposite charges on the aluminum electrodes attached to it. This process significantly boosts the overall triboelectric performance to achieve higher output voltage and current.

Based on the device model, a COMSOL simulation was modeled by studying electrostatic physics between the materials, and it could visualize the variation in the electric potential distributions on the surfaces of SAP powder and baking sheets for various positions of the diaper powders. The maximum vertical gap between the two baking sheets was set as 7 mm, and the variation in the vertical distance between the lower baking sheet and the powder was considered as a variable parameter “y” so that the powder could move between the vertical distances from 0 to 7 mm. The potential distributions at different y values, 0.5 mm, 3.5 mm, and 6.25 mm, are illustrated in Figure 6G. This COMSOL simulations analysis of the model was used to validate the experimental results discussed in the upcoming sessions.

### Electrical performance

The electrical performance of DW-TENG was rigorously characterized to evaluate its capability for energy harvesting and self-powered applications. The primary focus was on quantifying the electrical parameters, including voltage (V), current (I), and power output (P), under various operating conditions. The device was connected to a linear motor, and electricity generated across the terminals of DW-TENG under mechanical agitations at different operating frequencies of the linear motor was observed.

**Figure 7:**
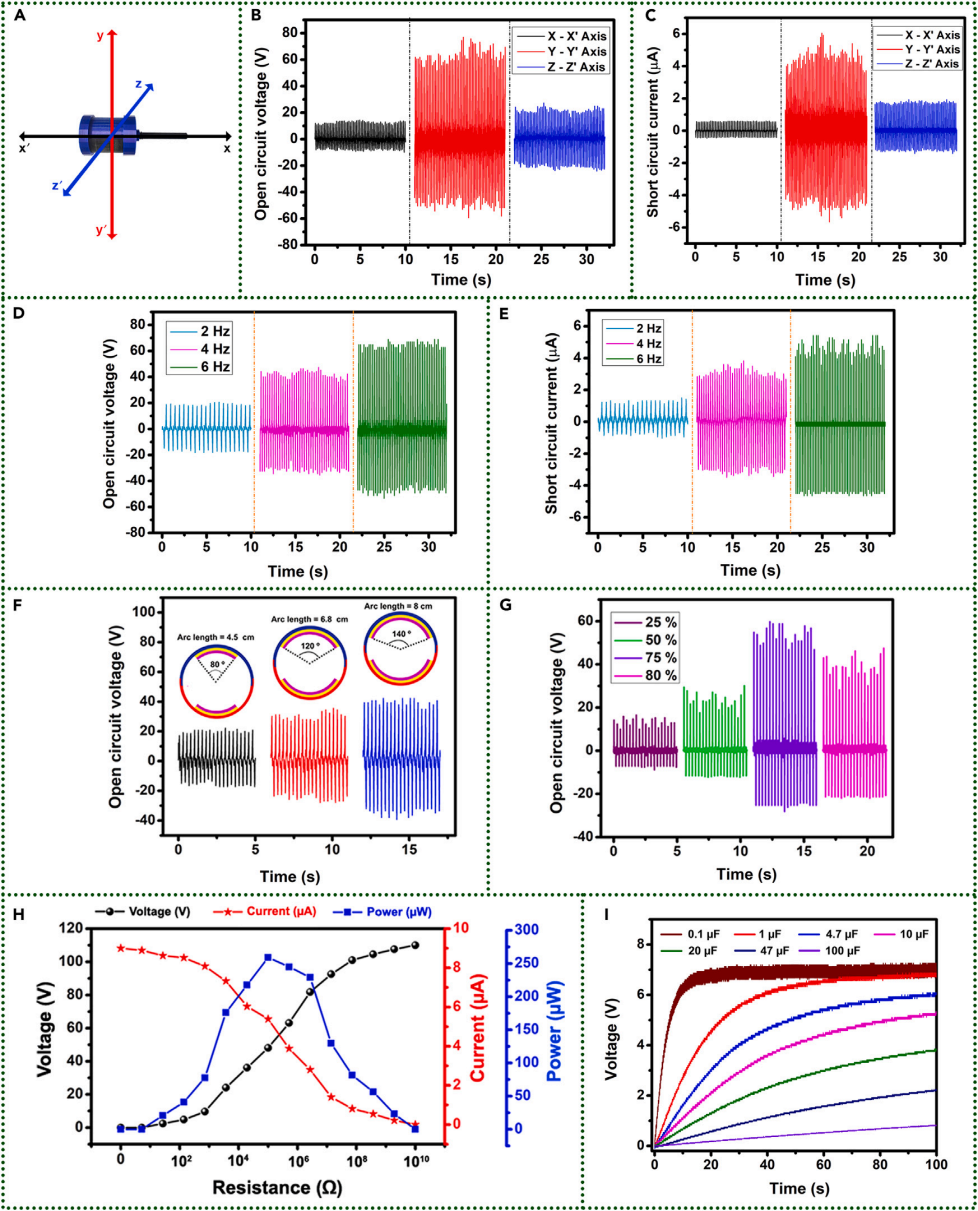
Electrical performances of DW-TENG (A) Axis orientations. (B and C) Voltages and currents obtained during shaking along different axes at 6 Hz frequency. (D and E) Voltages and currents obtained during shaking along y-y′ axis at different frequencies. (F and G) Voltages during shaking along y-y′ axis at 4 Hz shaking frequency with different arc lengths of baking sheet and fill ratio of dried SAP powder. (H) Load analysis. (I) Capacitor charging test.

As part of device optimization, the electrical performance of DW-TENG was primarily tested by shaking it along the x-x′, y-y′, and z-z′ axes, as shown in Figure 7A, at a controlled operational frequency of 6 Hz. The DW-TENG was fixed on a linear motor, and the shaking amplitude was maintained at 6 cm along each axis. The shaking axis was adjusted by orienting the DW-TENG in different directions. The voltage and current generated while shaking the device along each axis were observed and plotted, as shown in Figures 7B and 7C. A Keithley Electrometer 6514 was used to measure the voltage and current outputs, and data were recorded as a function of time. DW-TENG shows the best electrical output of 110 V and 9 μA along y-y′ axis vibration due to the maximum contact area between triboelectric materials and the free movement of SAP powders between top and bottom baking sheets. At the same time, the voltage/current along the x-x′ and z-z′ axes were 15 V/1 μA and 42 V/3 μA, respectively. Results indicated the capability of DW-TENG for scavenging energy along multi-axis vibrations.

The electrical performances of the DW-TENG along the y-y′ axis were tested and analyzed at different operational frequencies of 2 Hz, 4 Hz, and 6 Hz, as shown in Figures 7D and 7E. The corresponding shaking amplitudes for these frequencies were maintained at 6 cm along each axis. The results show that voltage and current have a linear relationship with operating frequency. As the frequency was increased to 2 Hz, 4 Hz, and 6 Hz, the corresponding peak-to-peak voltages and currents increased to 35 V/2 μA, 72 V/6 μA, and 110 V/9 μA.

The impact of the geometric size of the baking sheet and quantity of SAP powder on the device performance was systematically studied by varying the central angle of the baking sheet arc, adjusting the fill ratio of dried SAP powder inside the cylinder, and measuring the device’s voltage at a shaking frequency of 4 Hz along y-y′ axis. The DW-TENG’s response to different baking sheet arc central angles of 80º, 120º, and 140º was evaluated with corresponding arc lengths of 4.5 cm, 6.8 cm, and 8 cm, respectively, by keeping the fill ratio of the SAP powder as 75%. Figure 7F depicts the observed peak-to-peak voltages as 36 V, 54 V, and 73 V for the respective angles. The increasing trend aligns with expectations, as a larger arc facilitates more significant triboelectric interactions, resulting in higher voltage outputs. This behavior underscores the dependence of the device’s response on the area of contact between the SAP powder and the baking sheet.

Further, maintaining a fixed baking sheet arc length of 8 cm and central angle of 140º, the DW-TENG’s electrical performance was analyzed under varying fill ratios of dried SAP powder: 25%, 50%, 75%, and 80%. The corresponding peak-to-peak voltages were measured as 20 V, 35 V, 79 V, and 60 V, respectively (Figure 7G). The observed increasing trend up to a fill ratio of 75% suggests that the response of the DW-TENG is intricately linked to the area of contact between the SAP powder and the baking sheet. Beyond 75%, the free movement of the SAP powder within the cylinder was obstructed due to the tight occupancy. The restrictive environment led to decreased mobility of the SAP particles, limiting their ability to induce the desired charge separation essential for effective energy harvesting. While 6 Hz corresponds to the maximum electrical output, the tests in Figures 7F and 7G were done at 4 Hz to demonstrate the device’s performance at different operational conditions and help to illustrate the linear relationship between frequency and electrical output.

Load analysis of the device at 6 Hz frequency was tested, as shown in Figure 7H, to understand the variations in generated voltage and current for a range of load resistances, from 1 to 10^10^ Ω. The instantaneous values of the measured voltage and current were multiplied to get the instantaneous power output of the device. It is observed that as the load resistance increases, the output voltage increases from 0 to 110 V, while the current decreases from 9 μA to 0 A, which follows Ohm’s law (V = IR). Maximum power of 259.15 μW was noticed at 10^5^ Ω load resistance.

Finally, a capacitor charging analysis of DW-TENG was performed to observe how the device maintains its ability to charge capacitors during repeated operation, which is essential for determining the feasibility of using DW-TENG in practical applications. Capacitors with values of 0.1 μF, 1 μF, 4.7 μF, 10 μF, 20 μF, 47 μF, and 100 μF were connected to DW-TENG through a bridge rectification circuit. The charging and discharging of these capacitors were observed for continuous shaking of the device at 6 Hz frequency, as shown in Figure 7I. The results demonstrated that the charging times increased with the capacitance values, with the 0.1 μF capacitor charging up to 7 V within 3 s and larger capacitors charging at correspondingly slower rates. Videos S1 and S2 show the capacitor charging and real-time application using the 0.1 μF capacitor, respectively, where Video S1 shows the charging of the 0.1 μF capacitor and Video S2 demonstrates its use as a power source for a digital thermometer.

Video S1. Capacitor charging test by shaking DW-TENG, related to Figure 7

Video S2. Powering a digital thermometer by shaking DW-TENG, related to Figure 2

### Real-time applications

The DW-TENG demonstrates its versatility through two primary real-time applications: a rotational wind energy harvester and a self-powered anemometer. These applications highlight the practical utility of the device in harnessing ambient mechanical wind energy for sustainable energy generation and environmental monitoring.

### Rotational wind energy harvester

**Figure 8:**
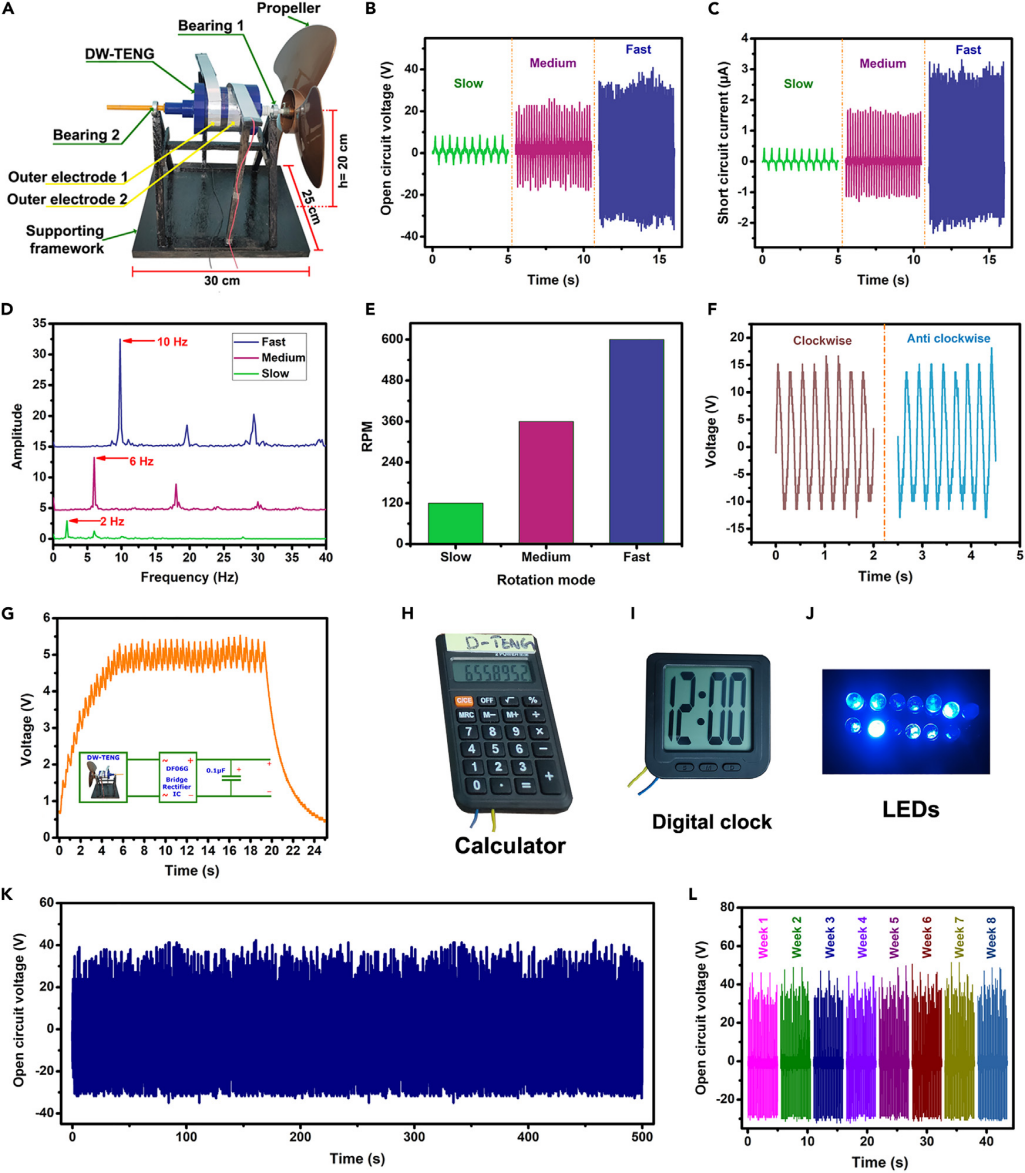
DW-TENG as rotational energy harvester (A) Device prototype. (B and C) Voltage and current outputs corresponding to slow, medium, and fast rotations. (D) Frequency data obtained by the FFT of the voltages from slow, medium, and fast rotations. (E) RPM for slow, medium, and fast rotations. (F) Polarity shift observed during clockwise and anti-clockwise rotations. (G) Capacitor charging performance at 360 RPM. (H–J) Powering of calculator, digital clock, and series-connected LEDs. (K–L) Stability test at 600 RPM for continuous 500 s and successive eight weeks.

The DW-TENG represents an innovative approach to energy harvesting, particularly in the realm of rotational energy from wind exposure. In this study, the real-time application of the DW-TENG was demonstrated by mounting it on a supporting framework with the assistance of two ball bearings fixed on the holders attached to the upper and bottom caps, as shown in Figure 8A. A propeller was affixed to one end of the holder, allowing rotation of DW-TENG during wind exposure. Electrode connections were established by extracting low gauge electrode terminals from the inner aluminum electrodes through small holes in the DW-TENG’s half-cylinders, and these terminals were then connected separately to two parallel aluminum layers surrounding the cylinder. Outer aluminum connections from the supporting framework were linked to these parallel aluminum layers, enabling the efficient transfer of DW-TENG output to external circuits.

In the experimental exploration of the DW-TENG as a rotational wind energy harvester, a series of tests were conducted to evaluate its performance under various conditions. A speed-adjustable table fan was positioned 2 m away from the DW-TENG’s propeller, simulating wind conditions. Due to the rotation of DW-TENG, SAP powder inside the cylinder slides over through two baking sheets, and due to freestanding operation between the inner SAP power and the baking sheet, a potential difference was created between the two electrodes attached bottom to the baking sheets. The device exhibited voltage and current outputs corresponding to slow (13 V/0.7 μA), medium (39 V/2.7 μA), and fast (64 V/4.9 μA) speeds (Figures 8B and 8C). This observation underscores the DW-TENG’s adaptability to different wind speeds, which is essential for real-world applications. Fast Fourier transform (FFT) signal analysis provided frequency data for the voltage signals. The frequencies corresponding to slow, medium, and fast speed modes were found to be 2 Hz, 6 Hz, and 10 Hz, respectively (Figure 8D). Converting these frequencies to rotations per minute (RPM) yielded values of 120, 360, and 600 RPM (Figure 8E). This dual functionality positions the DW-TENG as both an energy harvester and a rotational speed sensor.

The DW-TENG’s electrode polarity was confirmed by observing a 180º phase shift between voltage waveforms during clockwise and anti-clockwise rotations (Figure 8F). This phase shift validation is crucial for precise integration into electronic systems. The DW-TENG showcased efficient capacitor charging capabilities. Rotating the device at 360 RPM charged a 0.1 μF capacitor to 5 V within 5 s (Figure 8G). This rapid charging potential highlights the DW-TENG’s suitability for energy storage applications. The energy stored in the charged capacitor was effectively utilized to power practical electronic devices such as a calculator, a digital clock, and 13 series-connected LEDs, as shown in Figures 8H–8J and Videos S3, S4, and S5. This real-time demonstration emphasizes the DW-TENG’s potential for powering low-energy electronic gadgets.

Video S3. Rotational energy harvesting: Powering calculator, related to Figure 8

Video S4. Rotational energy harvesting: Powering digital clock, related to Figure 8

Video S5. Rotational energy harvesting: Powering 13 series connected LEDs, related to Figure 8

A comprehensive stability test was conducted to evaluate the stability and durability of the DW-TENG rotational energy harvester. The DW-TENG underwent continuous rotation at 600 RPM for 500 s to assess its performance under prolonged operational conditions. The output voltage was consistently measured throughout the test, revealing a peak-to-peak voltage of 64 V (Figure 8K). This result indicates the device’s robust stability and sustained performance even during continuous high-speed rotation.

Additionally, the stability test was extended over eight weeks, with separate measurements taken each week, as shown in Figure 8L. Surprisingly, the DW-TENG exhibited a remarkable consistency in output performance, maintaining a peak-to-peak voltage of 64 V throughout each week. This observed stability over an extended time frame underscores the device’s long-term reliability and durability. These findings enhance the confidence in the DW-TENG’s performance in real-world applications as a renewable energy harvester, emphasizing its potential for sustainable energy harvesting over prolonged periods. It is crucial in establishing its practical applicability and reliability.

### Self-powered anemometer

**Figure 9:**
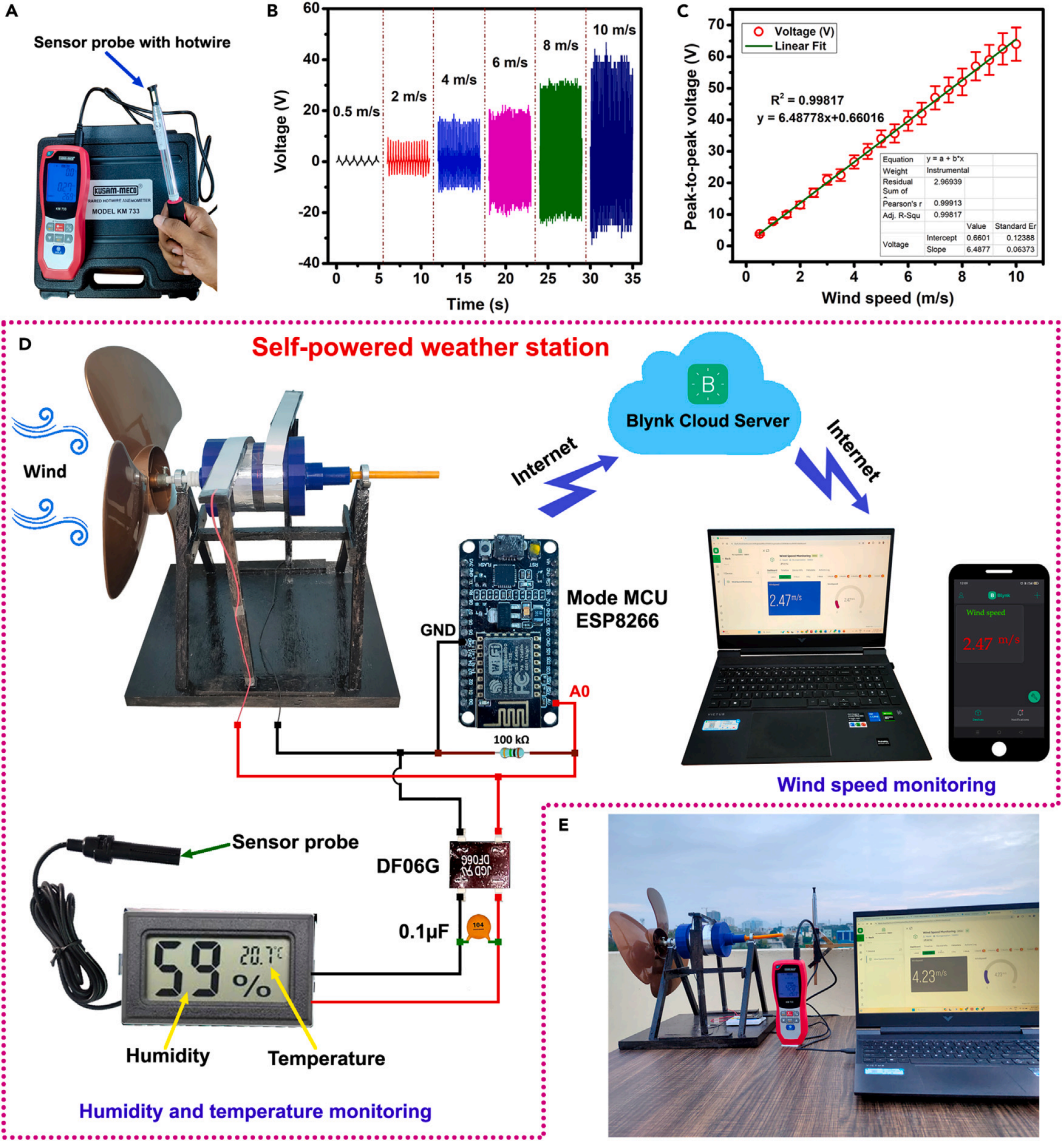
DW-TENG as self-powered anemometer (A) Infrared hotwire anemometer. (B) Voltage data corresponds to various wind speeds. (C) Linear fitting regression analysis of wind speed versus voltage plot. (D) Real-time wind speed, humidity, and temperature monitoring. (E) Practical outdoor testing of wind speed monitoring.

The successful demonstration of the DW-TENG rotational energy harvester extended its application as a self-powered anemometer to monitor real-time wind speed further by finding a mathematical relationship between the voltage generated from the rotating DW-TENG and the wind speed. A calibrated IR hotwire anemometer (KUSAM-MECO, Model KM 733), as shown in Figure 9A, was used to measure the wind speed placed near the propeller. At the same time, a digital storage oscilloscope was used to measure the voltage signals generated by the rotating DW-TENG. The IR hotwire anemometer is a traditional instrument for measuring wind speed in various applications. It has a sensor probe that houses the hotwire or hot filament heated to a constant temperature. The velocity of the air is then determined based on the cooling effect on the wire. The table fan speed was raised to get IR hotwire anemometer readings from 0.5 m/s to 10 m/s, and the corresponding voltage outputs from the rotating DW-TENG were recorded and analyzed.

The voltage data corresponding to wind speeds 0.5, 2, 4, 6, 8, and 10 m/s are shown in Figure 9B. It could notice a linear relationship between wind speed and voltage, suggesting that as wind speed increases or decreases, there is a proportional change in the voltage generated by the DW-TENG anemometer. A linear fitting regression analysis was conducted, as in Figure 9C, to obtain a fitting straight-line with equation y = 6.48778x+0.66016 with a coefficient of determination, R^2^_=_ 0.99817. The variables x and y represent the wind speed measured by the IR anemometer and the corresponding voltage measured by the oscilloscope, respectively. The R^2^ value is a measure of the goodness of linear fit. The equation indicates that for every unit increase in wind speed (x), the voltage (y) increases by a factor of 6.48778, with an additional constant value of 0.66016. This is a common form of a linear equation, where the coefficient 6.48778 represents the slope of the line, and the constant term 0.66016 is the y-intercept.

Later, this relation was used to program a NodeMCU ESP8266 microcontroller to find wind speed directly from the voltage generated by the DW-TENG rotation and to display the live wind speed values through a Blynk IoT cloud platform with the support of the internet. A NodeMCU ESP8266 microcontroller was connected to the DW-TENG rotational energy harvester outputs and interfaced with Blynk cloud support for IoT functionality, as shown in Figure 9D and Videos S6, and S7. This setup allowed for remote wind speed monitoring through the Blynk application web dashboard, accessible via computer or mobile phone from any location with internet access.

Video S6. Real-time wind speed monitoring, related to Figure 9

Video S7. Comparing wind speed data with the IR anemometer data, related to Figure 9

At the same time, the AC sinusoidal voltage output from the DW-TENG rotational energy harvester was rectified and filtered using a DF06G bridge rectifier and a 0.1 μF capacitor (Figure 9D). The resulting DC voltage was utilized to power a QBM Mini digital thermometer hygrometer. This thermometer hygrometer sensor displays the real-time atmospheric temperature and percentage of the relative humidity. It could successfully demonstrate the DW-TENG’s ability to monitor wind speed as a self-powered anemometer (Figure 9E) and act as an autonomous power source for a digital thermometer hygrometer. The setup explored its potential as a self-powered weather station by measuring real-time wind speed, humidity, and temperature (Video S8). The interconnected data streams provided a comprehensive overview of environmental conditions. The DW-TENG generates electrical power from ambient mechanical wind energy without the need for any external power source. In this sense, the setup constitutes a self-powered operation and enables continuous monitoring of environmental conditions using the harvested energy.

Video S8. Self-powered weather station, related to Figure 9

### Conclusion

The DW-TENG represents a significant advancement in sustainable weather monitoring and recycling practices by utilizing SAP gels from discarded diapers and non-stick baking sheets. With an estimated 250 million single-use diapers discarded daily worldwide, the calculated power generation potentials from globally disposed diapers could reach 12.95 kW daily, 388.72 kW monthly, and 4.72 MW yearly, as illustrated in Figure S4. Future work could extend this concept to develop multi-directional rotating energy harvesters, enhancing the range and efficiency of environmental monitoring applications, such as air quality and water pollution sensors. Integrating machine learning techniques could further optimize these systems for smart city applications, paving the way for more intelligent and sustainable urban environments. These advancements would further amplify the impact of the DW-TENG, solidifying its role as a critical technology in addressing global sustainability goals.

### Limitations of the study

While pioneering in the application of recycled diaper waste in TENG technology, this study acknowledges several limitations. The scalability of the process and the efficiency of energy conversion over larger or varied batches of diaper waste have not been comprehensively tested. Further, the environmental impact and feasibility of widespread implementation of this recycling process remain theoretical and require extensive field testing and environmental assessment. The long-term durability and performance consistency of the TENG devices under different environmental conditions are also areas that need further exploration.

## Resource availability

### Lead contact

Arunkumar Chandrasekhar (arunkumar.c@vit.ac.in).

#### Materials availability

Materials used in the study are commercially available.

#### Data and code availability

• All data reported in this paper will be shared by the lead contact upon reasonable request.
• No new code was generated during the course of this study.
• Any additional information required to reanalyze the data reported in this paper is available from the lead contact upon reasonable request.

### Method details

The study employed a series of systematic methodologies to fabricate and characterize the DW-TENG toward developing a self-powered weather station. The setup explores the triboelectric properties of recycled SAP powders and baking sheets to generate electricity through contact electrification.

#### Sterilization

Dried SAP powder and baking sheets were sterilized before reuse by soaking them individually in 70% ethanol for 30 min, heating them for 20 min at 70°C in a hot air oven, and then exposing them for another 30 min to long-wave UV light with a wavelength of 365 nm.^65^^,^^66^^,^^67^

#### Sterility testing

A direct inoculation sterility testing was performed on the sterilized dried SAP powders and baking sheet in an aseptic room to critically assess whether the sterilized materials were free from contaminating viable microorganisms and to confirm the reusability of the materials toward the DW-TENG fabrication. Initially, a sterile nutrient broth culture media was prepared, and the test samples from both materials were incubated at 37°C and monitored continuously for 14 days.^68^ Positive and negative controls were used to compare the test results of the materials. In positive control, the medium with added known organisms (*Escherichia coli*) but without test samples was used to confirm the culture media was capable of microorganism growth. Similarly, a medium without adding both organism and test samples was used in the negative control to verify that any microorganism did not contaminate the culture medium. After 14 days of incubation, turbidity was visualized only in the positive control but not in the negative control or the test sample medium. The sterility test results of the test samples were compared with the positive and negative control samples, and the test negativity on both the sterilized materials was confirmed, as shown in Figure S5. The lack of turbidity in the test sample media validates the absence of viable microorganisms over the materials. After the sterility test, the naturally dried SAP powder and the baking sheet were reused as integral components of the DW-TENG for energy harvesting and self-powered anemometer application.

#### Proportion

The DW-TENG device was fabricated using approximately 80% SAP gel powder from discarded diapers and 20% baking sheets. Specifically, 150 g of SAP gels and 37.5 g of baking sheets were used in the device.

#### Molecular weight

The molecular weight of the SAP gels, primarily composed of sodium polyacrylate, is approximately 94.05 g/mol per repeating unit. The baking sheets are coated with PTFE, which has a molecular weight of approximately 100.02 g/mol per repeating unit. The molecular weight calculation for the SAP gels is based on the repeating unit of sodium polyacrylate (C_3_H_3_NaO_2_), where the molecular weights of the individual elements are as follows: Carbon (C) is 12.01 g/mol, Hydrogen (H) is 1.01 g/mol, Sodium (Na) is 22.99 g/mol, and Oxygen (O) is 16.00 g/mol. Adding these together gives a molecular weight of 94.05 g/mol for one repeating unit. Similarly, the molecular weight calculation for PTFE is based on the repeating unit of PTFE (C_2_F_4_), with Carbon (C) being 12.01 g/mol and Fluorine (F) being 19.00 g/mol, resulting in a molecular weight of 100.02 g/mol per repeating unit.

#### Mechanical properties

The SAP gels exhibit high water absorption capacity and swellability, with the ability to absorb up to 400 g of water per gram of dry polymer. As observed from SEM images, the shape of the SAP particles shows irregular granular structures. The size of these particles varies, but they generally measure around 300 μm. The density of the SAP, calculated from mass and volume measurements, is approximately 0.788 mg/mL. The baking sheets are coated with PTFE, and their tensile strength was measured to be 21.85 MPa. The baking sheets also demonstrate excellent flexibility and rollability, with the ability to bend to 90º, 180º, and roll into a cylindrical shape, as shown in Figure S6.

## STAR★Methods

### Key resources table

**Table:** 

REAGENT or RESOURCE	SOURCE	IDENTIFIER
**Other**		

Diapers	Landfills	N/A
Baking Sheets	Food industry waste	N/A
Aluminum Foil	Commercial Supplier	N/A
Low gauge copper wires	Commercial Supplier	N/A

**Software and algorithms**		

Origin 2022	OriginLab	N/A
COMSOL Multiphysics 6.0	COMSOL	N/A
SOLIDWORKS	SOLIDWORKS Corp	N/A
Blynk IoT Platform	Blynk	N/A

## Supplemental information

Document S1. Figures S1‒S6
