# Supplementary material for: Recycling of diaper wastes for a triboelectric nanogenerator-based weather station
Source: iScience. 2024 Jul 31;27(9):110627. doi: 10.1016/j.isci.2024.110627 (PMC11369379; doi:10.1016/j.isci.2024.110627)
Supplement: Document S1. Figures S1‒S6 [file mmc1.pdf]

iScience, Volume *27*

Supplemental information

Recycling of diaper wastes for a triboelectric nanogenerator-based weather station

Sayyid Abdul Basith, Ananthakumar Ramadoss, Gaurav Khandelwal, George Jacob, and Arunkumar Chandrasekhar

Environmental impact of diaper recycling process

The recycling process for diapers involves several steps, including collection, sterilization, separation of materials, and conversion into useful components for the triboelectric nanogenerator. Each of these steps has potential environmental impacts, which we have evaluated as follows:

- The collection of diapers reduces the volume of waste sent to landfills.
- The sterilization process uses autoclaving or chemical treatment to ensure that the diapers are free from pathogens. Autoclaving is energy-intensive but effective in minimizing the environmental hazards associated with untreated diaper waste. Chemical treatments may involve the use of disinfectants, which are managed to prevent environmental contamination.
- Diapers are mechanically separated into components such as super-absorbing polymer (SAP) gels, plastic films, and nonwoven fabrics. This mechanical process is designed to be energy-efficient and minimizes the release of microplastics into the environment. Additionally, the plastic films and nonwoven fabrics, composed of polyethylene or polypropylene, offer possibilities for future utilization as triboelectric materials for TENG fabrication, although this was not explored in the current work.
- The SAP gels are processed into materials for the TENG. This step involves minimal chemical processing, reducing the risk of environmental pollution. The use of recycled materials significantly lowers the demand for virgin raw materials, thereby conserving natural resources.
- The TENGs created from recycled diaper materials are designed for long-term use. At the end of their lifecycle, the components can be further recycled or disposed of with minimal environmental impact compared to traditional electronic waste.

The environmental benefits of this process are significant. Recycling diapers diverts a considerable amount of waste from landfills, where diapers can take hundreds of years to decompose. The use of recycled materials conserves raw materials and reduces the energy consumption and emissions associated with the production of new materials. By integrating waste materials into useful products, the process contributes to a reduction in overall carbon emissions and environmental pollution.

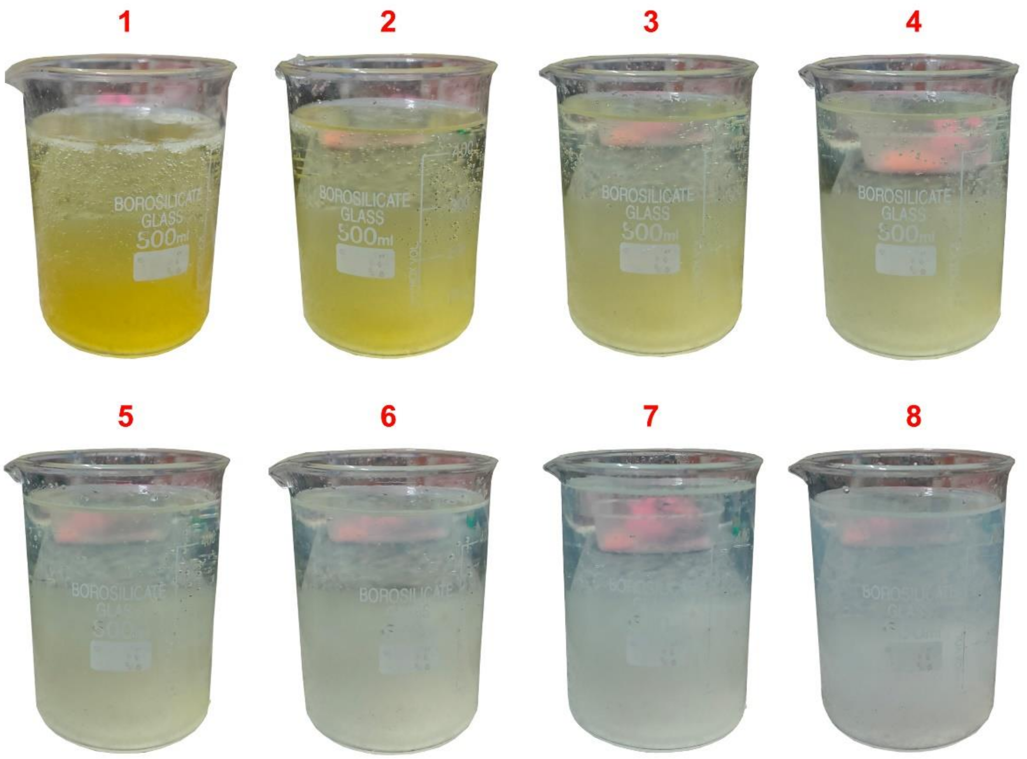

Figure S1. Cleaning test of urinated super-absorbing polymer gel with multiple rinsing with water, Related to Figure 3

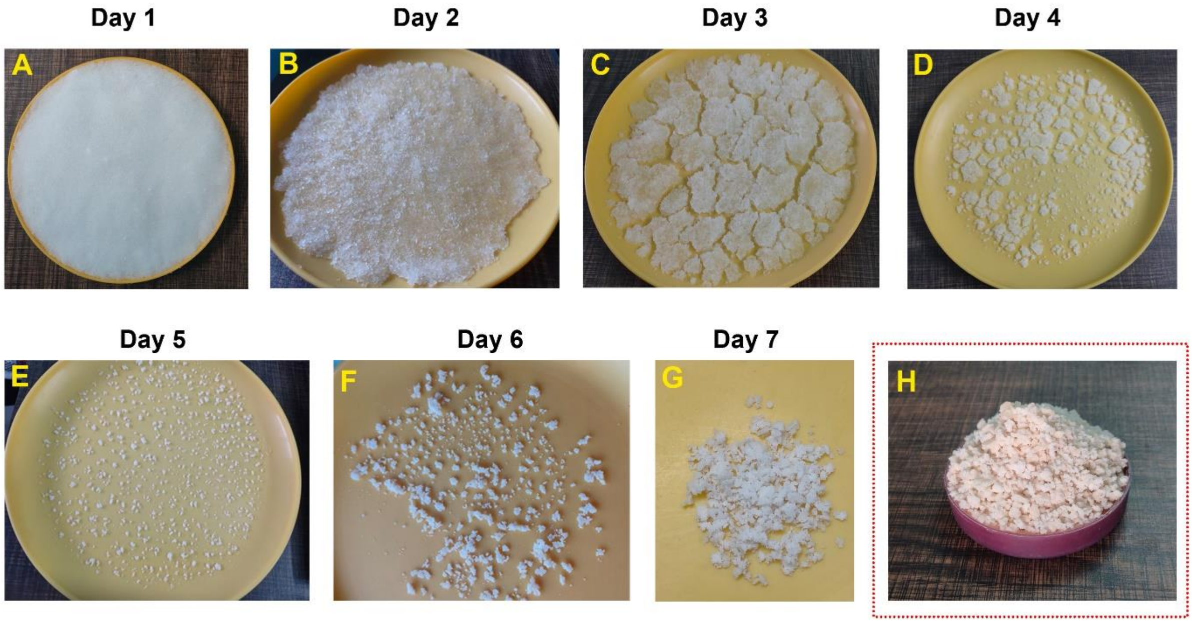

Figure S2. Natural drying of SAP gel, Related to Figure 3
(A-G) Day-by-day analysis of natural drying for seven days.
(H) Final collected dried SAP powder.

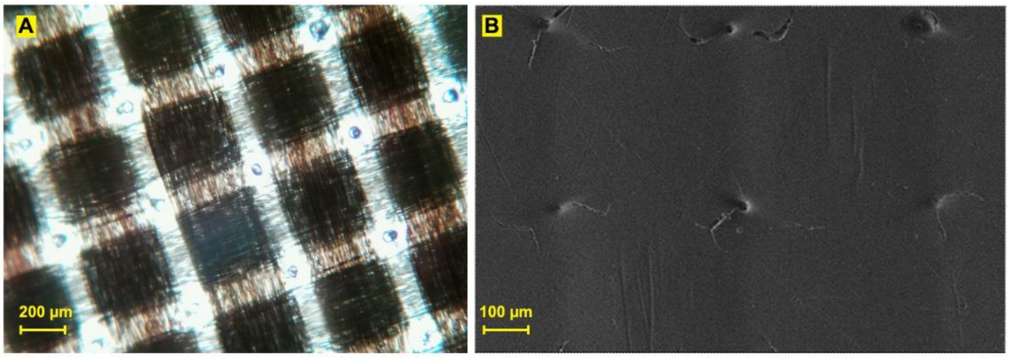

Figure S3. Microscopic analysis of baking sheet, Related to Figure 4
(A) High-resolution optical microscopic visual.
(B) Surface morphology analysis using SEM.

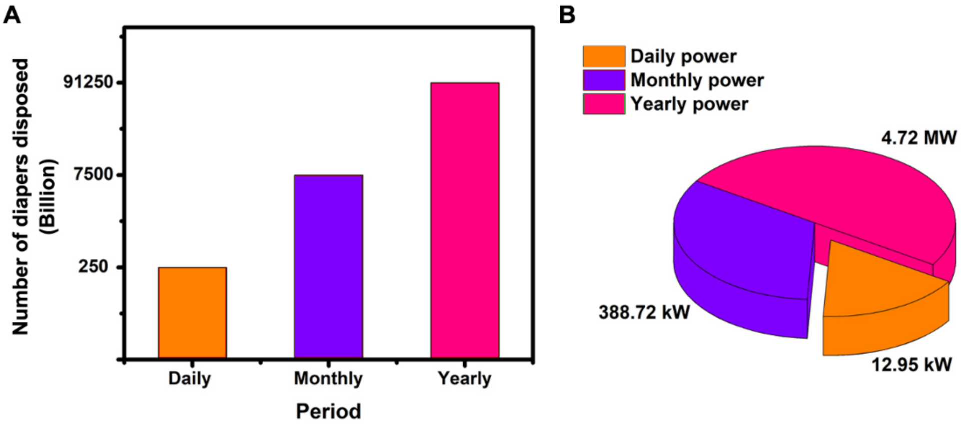

Figure S4. Endless possibilities of recycling diapers, Related to Figure 9
A) Number of diapers dispersed daily, monthly, and yearly.
B) Electric power generation potentials from the disposed diapers.

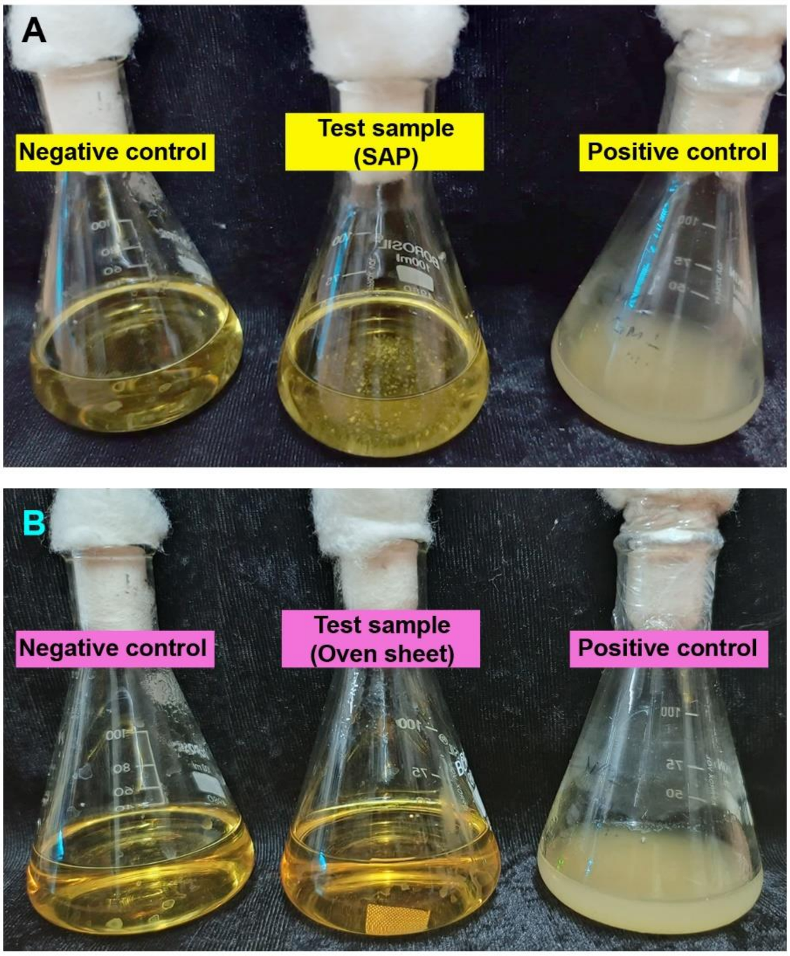

Figure S5. Sterility test results after 14 days of incubation, Related to STAR Methods
(A) Naturally dried SAP powder.
(B) Baking sheet.

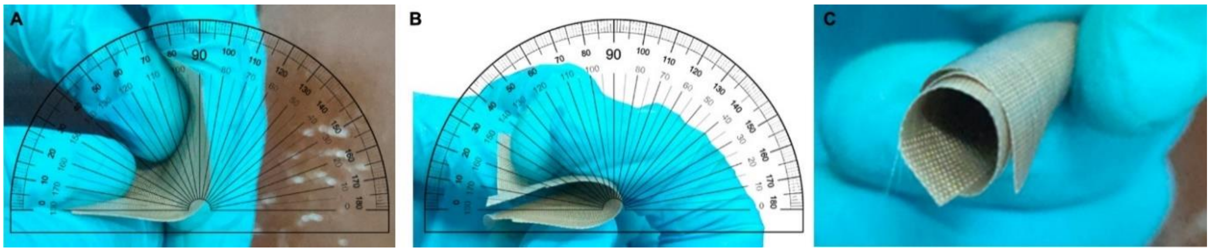

Figure S6. Flexibility and rollability of the baking sheets, Related to STAR Methods
(A) Bending to 90 degrees.
(B) Bending to 180 degrees.
(C) Rolling into a cylindrical shape.
